# Which Factors Determine Metal Accumulation in Agricultural Soils in the Severely Human-Coupled Ecosystem?

**DOI:** 10.3390/ijerph13050510

**Published:** 2016-05-17

**Authors:** Li Xu, Shanshan Cao, Jihua Wang, Anxiang Lu

**Affiliations:** 1 Beijing Research Center for Agricultural Standards and Testing, Beijing Academy of Agriculture and Forestry Sciences, Beijing 100097, China; xuliforever@163.com (L.X.); shanshancao1413@sina.com (S.C.); jhwang1995@sina.com (J.W.); 2 Beijing Municipal Key Laboratory of Agriculture Environment Monitoring, Beijing 100097, China

**Keywords:** heavy metal, land use, soil type, urbanization, interaction

## Abstract

Agricultural soil is typically an important component of urban ecosystems, contributing directly or indirectly to the general quality of human life. To understand which factors influence metal accumulation in agricultural soils in urban ecosystems is becoming increasingly important. Land use, soil type and urbanization indicators all account for considerable differences in metal accumulation in agricultural soils, and the interactions between these factors on metal concentrations were also examined. Results showed that Zn, Cu, and Cd concentrations varied significantly among different land use types. Concentrations of all metals, except for Cd, were higher in calcareous cinnamon soil than in fluvo-aquic soil. Expansion distance and road density were adopted as urbanization indicators, and distance from the urban center was significantly negatively correlated with concentrations of Hg, and negatively correlated with concentrations of Zn, and road density was positively correlated with Cd concentrations. Multivariate analysis of variance indicated that Hg concentration was significantly influenced by the four-way interaction among all factors. The results in this study provide basic data to support the management of agricultural soils and to help policy makers to plan ahead in Beijing.

## 1. Introduction

Soil environments are a critical interface between the earth, the atmosphere and water. Soil environments are also habitats for a rich variety of organisms and provide many regulating and supporting services [1]. Because the processes involved in soil formation are very slow, and recovery strategies are very complex and expensive, soil can be considered a non-renewable resource [2]. Soil contamination with heavy metals has been of worldwide concern [3]. Heavy metals are especially dangerous because of their persistence, toxicity and bioaccumulation characteristics [4], additionally, metals can be transferred from soil to the other ecosystem components, such as underground water or crops, and ultimately affect human health [5].

Urban ecosystems are severely impacted by human activities [6]. Agricultural soil was typically an important component of urban ecosystems, contributing directly or indirectly to our general quality of life [7]. Agricultural soil in urban ecosystems is affected not only by agricultural activities (in particular, the application of large amounts of fertilizer, manure and sludge), but also by urbanization and industrialization activities (for example, the output of industrial effluents, vehicle exhaust emissions, coal combustion, infrastructure development and tourism) [8,9,10]. The importance of assessing the accumulation concentrations and understanding how these factors influence heavy metal accumulation in agricultural soils is becoming increasingly important.

This study was conducted in the city of Beijing. Beijing is the political, economic and cultural center of China, with over 1000 years of history and more than 18 million residents [11]. Several studies targeting heavy metals have been conducted, including the analysis of soil metal concentrations at contaminated sites, soil heavy metal pollution in Beijing’s urban parks, and the spatial distribution and ecological risk of heavy metals in agricultural soil [12,13,14]. There is, however, limited information on how various soil and environmental factors affect the metal concentrations in agricultural soil in Beijing, in particular, information on how urbanization affects metal concentrations in agricultural soils and the interaction between different factors is lacking. Thus, elucidation of factors affecting heavy metal accumulation in agricultural soils is important for developing soil conservation and management strategies.

The objectives of this research were to: (1) determine concentrations of cadmium (Cd), copper (Cu), nickel (Ni), lead (Pb), chromium (Cr), zinc (Zn), arsenic (As) and mercury (Hg) in agricultural soils; (2) to elucidate how the environmental factors affect metals concentrations in soils; and (3) to elucidate the effects of the interactions between different factors on metal concentrations. The collected data will facilitate a better understanding of the accumulation status and factors affecting metal concentrations in agricultural soils in severely human-impacted ecosystems.

## 2. Materials and Methods

### 2.1. Site Description

Beijing, the capital of China, is a rapid developing city in northern China. Beijing is located in the northwest portion of the north China plain (E115.43°, N40.19°–E115.97°, N40.50°) and covers an area of approximately 1.6 × 104 km^2^ [15]. The city has a typical monsoon-influenced climate, characterized by hot, humid summers caused by the East Asian monsoon, and generally cold, windy, dry winters caused by the vast Siberian anticyclone. The mean annual precipitation is 470–660 mm, with approximately 60% of the precipitation occurring in July and August [16]. Its elevation ranges from 10 m in the southeast to 2030 m in the northwest. The primary type of soil in the in the plains area is fluvo-aquic soil (FAS) and calcareous cinnamon soil (CCS).

### 2.2. Soil Sampling/Pretreatment

**Figure 1:**
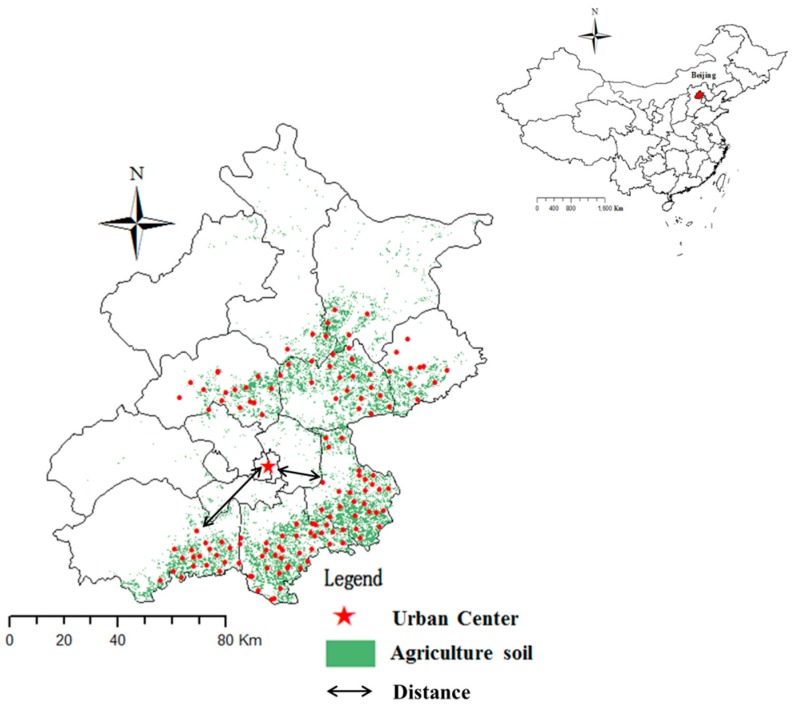
The agricultural soil sampling sites in the Beijing.

Soil sampling was conducted in 2011 at agricultural sites that were selected according to the distribution of agricultural soil. A total of 134 surface soil samples (each sample was a composite mixture of five sub-samples) were collected using a stainless-steel auger. Throughout the survey, a global positioning system (GPS) was used to locate the sampling sites (Figure 1). Soil was air-dried at room temperature, and each soil sample was divided into two parts. One part was crushed in an agate mortar to pass through a 2.0 mm mesh nylon sieve for the determination of pH, and another part was ground to pass through a nylon 100-mesh sieve for the determination of metals and organic matter.

### 2.3. Metals Analysis

To determine the total contents of Pb, Ni, Cu, Zn and Cd, soil was digested with HCl, HNO_3_, HF and HClO_4_ [17]. The digestion for determination of As and Hg used aqua regia (3:1 HCl–HNO_3_) [17]. Concentrations of Pb, Ni, Cu, Zn, and Cd in the digestion solution were determined by Atomic Absorption Spectrophotometry (AA-6300, Shimadzu, Kyoto, Japan). Concentrations of As and Hg in the digestion solution were determined by Atomic Fluorescence Spectrophotometry (AFS-830, Titan, Beijing, China). Soil standard reference materials (GSS-1 and GSS-2) obtained from the Center of National Standard Reference Material of China was analyzed as part of the quality assurance and quality control procedures.

The recovery rates of Cu, Zn, Ni, Pb, As, Cd and Hg were 95%–105%, 96%–108%, 92%–102%, 92%–98%, 101%–103%, 98%–103% and 95%–98%, respectively. The detection limits were 0.04 mg/kg for Cu, 0.01 mg/kg for Cd and As, 0.05 mg/kg for Ni, Pb and Zn, and 0.002 mg/kg for Hg. The metal concentrations for reference materials were within the range of the certified value and the replicate analysis of each batch of samples showed that the analytical precision was within 10% variability. Samples were carefully handled to avoid introduction or loss of trace elements during preparation and analysis. All materials used during analytical determinations were kept in Teflon or other metal-free containers.

### 2.4. Statistical Methods

Statistical analyses were conducted with Microsoft Excel (IBM Corpotation, New York, NY, USA) and SPSS 13.0 statistical software (IBM Corpotation, New York, NY, USA). The distribution of metals concentrations were tested with the Kolmogorov-Smirnov method to determine if they approximated the normal probability distribution. Statistical evaluation was performed by analysis of variance (ANOVA), multivariate analysis of variance (MANOVA) and least significant difference (LSD) *post-hoc* tests. The level of significance was established at *p* < 0.05 (two-tailed).

## 3. Results and Discussion

### 3.1. Metal Concentrations in Agricultural Soil

Descriptive statistics of the heavy metals in the agricultural soils are presented in Table 1. The pH of the agricultural soils in the study area was between 6.28 and 8.34, with an average of 7.65. The mean concentration of Hg in soils was 0.07 mg·kg^−1^, dw. The Hg values had a coefficient of variation of 112% which was the largest of all the elements studied. This observation was the result of the heterogeneity of Hg contents, with a few locations having elevated concentrations. Mean concentrations of As, Cd, Cu, Hg, Ni, Pb and Zn were 8.38, 0.19, 24.4, 0.07, 24.5, 22.5 and 75.0 mg·kg^−1^, dw, respectively. Metal concentrations in agricultural soil occurred in the following order: Zn > Cu > Ni > Pb > As > Cd > Hg (Table 1). According to the Chinese Environmental Quality Standard for Soils [18], the guideline heavy metal concentration values in soil for agricultural products and human heath are 20, 0.6, 100, 1.0, 350, 250, 60 and 300 mg·kg^−1^ for As, Cd, Cu, Hg, Pb, Cr, Ni and Zn, respectively [19]. Only one sample contained Cd in excess of the guideline value; otherwise, all samples had metal concentrations below the guideline values.

### 3.2. Impacts of Urbanization on Soil Metals Accumulation

The Forbidden City is located at the center of Beijing, and urban sprawl extends outwards from this area [20]. We used the distance between each sample site and the Forbidden City as the urban expansion distance. The expansion distance was adopt as an indicator to represent the extent of urbanization of the sampling site. Urban expansion distance was significantly negatively correlated with concentrations of Hg (*p* < 0.01), and negatively correlated with concentrations of Zn (*p* < 0.05). Peng *et al*. [20] also found that distance from urban center was negatively correlated with concentrations of Zn in soil. A potential reason for the observed correlation between Zn and urban expansion distance is the presence of many historical wooden buildings in Beijing. Zn was historically used to produce red and yellow pigments, which were the predominant colors in such buildings [21]. In addition, Zn compounds in liquid form were also used as wood preservatives and fire retardants [22]. Over time, Zn used for such purposes would slowly be transferred into surrounding soils through physicochemical reactions and through erosion due to rain and wind. Additionally, coal, which is known to contain high concentrations of Hg and Zn [23,24], is used in smelting, electricity generation, and heating. Total coal consumption in Beijing is high; in 2010, consumption was about 7 × 10^7^ tons [25]. The more urbanization, the more consumption of coal, and lead to more Hg and Zn accumulated in soil.

Real-time traffic volume data for Beijing was unavailable, so we used road density to reflect the transportation capacity of local blocks, applying it as another urbanization indicator for the sampling sites (Supplementary Figure S1). A map of the road network was developed from remote sensing data, and road density was calculated using a search radius of 500 m in ArcGIS. The results in Table 2 indicate that the concentrations of Cd was correlated (*p* < 0.05) with road density, which is consistent with results of Peng *et al.* [20].

Vehicle exhaust and tire wear are two important sources of Cd [8], Zhang *et al.* [26] observed Cd accumulation in soil along the road in Guiyang, China. Wang *et al.* [27] found Cd accumulation in soil along the road in Beijing, China. In another study, vehicle exhaust was found to be an important source of Pb [23], However, there was no significant correlation between Pb concentrations and road density in our study area. This may be because the use of leaded gasoline in China was halted in 2001 [28].

### 3.3. Impacts of Land Use on Soil Metals Accumulation

**Figure 2:**
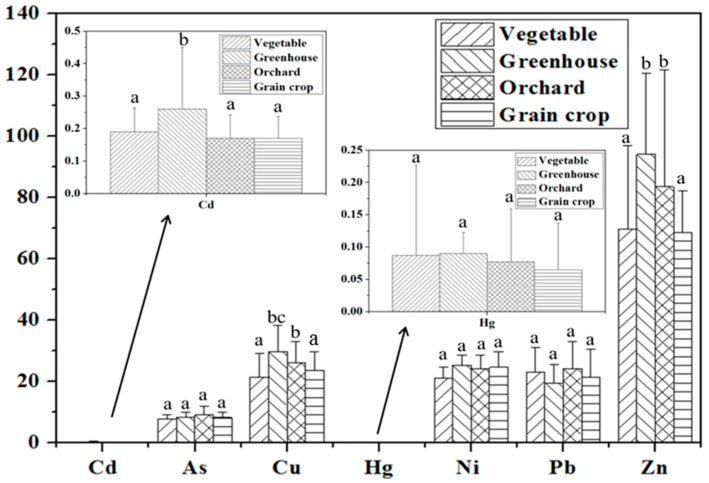
Heavy metals concentration in soils from different land use in soil (mg·kg^−1^). Land uses followed by different letters (a, b, c) are significantly different at *p* < 0.05.

Land use is an important factor that may affect metal distribution in soil because different land uses are characterized by different farming practices and soil properties, and may therefore be subject to different metal sources [8]. Soils collected from different land use patterns were analyzed to determine the effects of land use on metal accumulation. Four types of land use (vegetable land, greenhouse land, orchard land and grain crop land) were identified in agricultural soil. Greenhouse is a structure with walls and roof made chiefly of transparent material, such as glass or plastic, in which plants requiring regulated climatic conditions are grown. The results of ANOVA and descriptive statistics of metal concentrations in soils from four kinds of land uses are shown in Table 3 and Figure 2.

According to the results of ANOVA, there were significant differences between different land uses for Zn, Cu and Cd, and no significant differences for the rest metals (Table 3). Using *post-hoc* tests with LSD, it was found that Cd concentration in greenhouse land was significantly higher than other three kinds of land uses, Cu and Zn concentration in greenhouse land were significantly higher than grain crop land and vegetable land.

The highest average values for Cd (0.27 mg·kg^−1^, dw), Cu (28.2 mg·kg^−1^, dw), Hg (0.09 mg·kg^−1^, dw), Ni (25.4 mg·kg^−1^, dw) and Zn (95.1 mg·kg^−1^, dw) were found in the greenhouse land (Figure 2). Unlike the other land use types in our study, greenhouse land is subject to the high-intensity manure and fertilizer use, and is used for the continuous, year-round production of produce [3].

Some published literature also found metal accumulation in greenhouse land [29,30]. There are high concentrations of metals in fertilizer (especially phosphate fertilizers) and livestock manures [4,9,10]. The input amount of manure and fertilizer to greenhouse land was about 3907 kg/hm^2^/a (net) [31]. Elevated metals concentrations in greenhouse land use may come from the application of manure and fertilizer. The largest average values for Pb (24.7 mg·kg^−1^, dw) and As (9.20 mg·kg^−1^, dw) were observed in the orchard land, which is consistent with results of other studies [32,33]. Elevated Pb and As concentrations in orchard land has been shown to be most likely due to the use of As-based and Pb-based insecticides to control chewing insects [2,17]. The lowest mean values for As (7.72 mg·kg^−1^, dw), Ni (21.5 mg·kg^−1^, dw) and Cu (21.5 mg·kg^−1^, dw) were found in vegetable lands. The least mean values for Cd (0.18 mg·kg^−1^, dw), Zn (68.9 mg·kg^−1^, dw) and Hg (0.07 mg·kg^−1^, dw) were found in grain crop soils. Vegetable and grain crop soils contained lower metal concentrations because they received lower amounts of fertilizers and pesticides than greenhouse and orchard soils. The lowest mean values for Pb (19.6 mg·kg^−1^, dw) was found in the greenhouse land. All greenhouse production systems were covered with glass or plastics film, which can prevent atmospheric deposition of volatile metals, such as Pb [34].

### 3.4. Impacts of Soil Type on Soil Metals Accumulation

**Figure 3:**
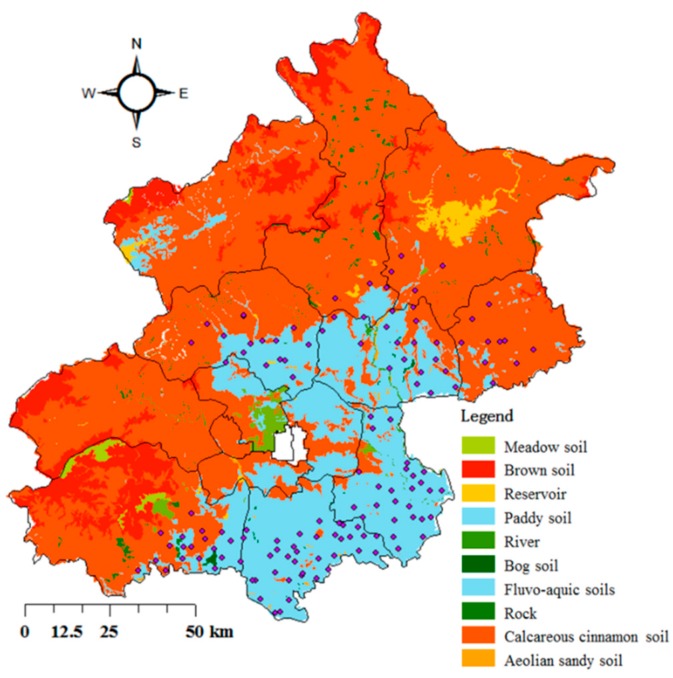
Soil types in the study area.

Soil type is expected to influence heavy metal accumulation in agricultural soils, because different soil types are composed of various soil parent matrixes, which can influence the levels of metals [7]. The two dominant soil types in the study area are CCS and FAS (Figure 3). Descriptive statistics of metal concentrations in different soil types are shown in Table 4. In general, metal concentrations varied widely between different soil types.

Mean concentrations of metals, except for Cd, were higher in CCS than in FAS in our study area. Xu *et al*. [17] found that the mean concentrations of Ni, Cr, As, Zn, and Cu were lower in FAS than in CCS in the watershed of the Guanting Reservoir, China. Chen *et al*. [35] found that soil type was a factor in determining the variation of metal concentrations in soils of Aragón, Spain. The background values of As, Cu, Ni and Zn in CCS were higher than that in FAS, which was consitant with accumulation status of these metals in agricultural soil. However, the remaining metals showed opposite results. This phenomenon means that the concentration of Cd, Pb and Hg in agricultural soil must be affected by other factors. We performed ANOVA to study the differences in metal concentration between different soil types (Supplementary Table S1); results indicated that there were no significant differences in metal concentrations between soil types.

### 3.5. Interaction of Different Factors for Heavy Metal

In addition to examining the effects of individual factors (land use type, urbanization, and soil type) on the accumulation of heavy metals in agricultural soil, we also examined the effect of the interaction between different factors on heavy metal concentrations in agricultural soil. For this analysis, we divided the sites into two clusters based on their distance from the urban center: suburbs (19.5–49.6 km away) and outer suburbs (49.6–79.7 km away). We also divided sites into two clusters based on road density: high road density (0.73–1.41 km·km^−2^) and low road density (0.08–0.73 km·km^−2^). We then performed a MANOVA of heavy metal concentrations in agricultural soils (Table 5). The MANOVA method was a very useful tool which has been used widely to clarify the interaction between factor for the accumulation of heavy metals in soil [37,38].

Results indicated that the As concentration is significantly influenced by the two-way interaction between soil type and distance from urban center, and by the three-way interaction between land use, soil type, and distance from urban center. There were no significant differences in as concentrations when only individual factors were considered. The Zn concentrations are significantly influenced by the two-way interaction between soil type and land use, and by the two-way interaction between road density and distance from urban center. The MANOVA results also demonstrated that Hg was the only element that is significantly influenced by the four-way interaction between all factors. This results mean that factors (land use, soil type and urbanization indicators) account for significant differences in Hg accumulation in the agricultural soils in our study area.

## 4. Conclusions

An extensive investigation of heavy metals in agricultural soils in the Beijing city was carried out. Mean concentrations of As, Cd, Cu, Hg, Ni, Pb and Zn were 8.38, 0.19, 24.4, 0.07, 24.5, 22.5 and 75.0 mg·kg^−1^, dw, respectively. The factors such as land use, soil type, and urbanization that influence the concentrations of these metals in agricultural soils were explored, as well as the effect of the interactions between factors. The urbanization indicated by distance from the urban center and road density affected Hg, Zn and Cd accumulation in agricultural soil. Coal consumption was the key source for Hg and Zn, the more urbanization, the more Hg and Zn were accumulated in agricultural soil. Vehicle exhaust and tire wearing were two important sources of Cd. The large application of manure and fertilizers resulted in elevated metal concentrations in greenhouses, especially for Cd, Cu and Zn. The concentrations of As, Cu, Ni and Zn in CCS were higher than that in FAS, while the opposite trend was observed for the rest of the metals. Hg was the only element which was significantly influenced by four-way interaction, which suggested land use, soil type and urbanization determined the Hg accumulation in the agricultural soils in Beijing city. This study gives a clear picture of the present accumulation of metals in agricultural soil in Beijing, and also clarified the important factors that controls metal accumulation in the agricultural soil, which would better the soil management in Beijing and benefit for policy makers to do further urban management and planning.

## Figures and Tables

**Table 1:** Basic descriptive data of heavy metals in agricultural soils in the Beijing.

	Min	Max	Mean	Range	Skew	Kurt	CV ^a^
As	4.05	18.3	8.38	14.2	0.98	3.43	24.3
Cd	0.06	0.77	0.19	0.71	3.12	14.2	50.3
Cu	8.40	49.8	24.4	41.4	0.97	1.18	28.4
Hg	0.01	0.67	0.07	0.66	5.18	32.7	112
Ni	9.60	36.4	24.5	26.8	0.00	−0.04	19.5
Pb	5.10	42.7	22.5	37.6	0.02	−0.84	39.1
Zn	32.9	260	75.0	227	3.53	21.8	33.4
pH	6.28	8.34	7.65	2.06	−1.17	1.53	5.10

^a^ CV: Coefficient of Variation (%).

**Table 2:** Correlations between heavy metals concentions and urbanization indicator.

Urbanization	Statistical Items	As	Cd	Cu	Hg	Ni	Pb	Zn
Distance from urban center	Correlation coefficient	0.16	−0.16	−0.06	−0.35 **	−0.08	−0.15	−0.21 *
	Sig. (2-tailed)	0.07	0.07	0.49	0.00	0.35	0.08	0.02
Road density	Correlation coefficient	−0.02	0.20 *	0.01	0.01	0.01	0.01	0.03
	Sig. (2-tailed)	0.86	0.02	0.91	0.96	0.97	0.94	0.77

** Correlation is significant at the 0.01 level (2-tailed); * Correlation is significant at the 0.05 level (2-tailed).

**Table 3:** ANOVA of heavy metals for different land uses in the agricultural soil.

	As	Cd	Cu	Hg	Ni	Pb	Zn
F	2.18	4.72	3.44	0.69	2.19	1.27	6.66
Significance	0.09	0.01 *	0.02 *	0.56	0.09	0.28	0.00 *

* Significance level of 0.05.

**Table 4:** Metal concentrations in different soil types in the agricultural soil.

Soil Type	N	Parameter	As	Cd	Cu	Hg	Ni	Pb	Zn
FAS	100	Min	4.32	0.09	8.40	0.01	9.60	5.10	32.9
		Max	12.9	0.77	43.9	0.58	43.8	39.5	159
		Mean	8.30	0.20	23.8	0.07	24.4	22.4	73.4
		SD	1.72	0.10	6.93	0.06	4.68	8.72	21.2
Background value of FAS ^a^	-	-	9.7	0.10	24.1	0.05	23.7	21.9	71.1
CCS	34	Min	4.05	0.08	13.6	0.01	13.9	9.20	47.2
		Max	18.3	0.31	43.8	0.67	36.4	42.7	260
		Mean	8.61	0.16	26.2	0.09	24.9	22.6	79.9
		SD	2.80	0.06	6.71	0.12	4.82	8.50	35.6
Background value of CCS ^a^	-	-	11.6	0.10	24.3	0.04	23.9	21.3	74.1

^a^ Background value: the background value of metals for two soil type were from [36].

**Table 5:** Multivariate ANOVA results for heavy metals in agricultural soils.

Group	Statistical Items	As	Cd	Cu	Hg	Ni	Pb	Zn
F1 * F2	F	2.55	1.68	1.27	0.33	1.45	0.45	6.45
	Significance	0.08	0.19	0.07	0.72	0.24	0.64	0.00
F1 * F3	F	1.43	0.34	0.33	1.67	1.56	1.43	0.63
	Significance	0.24	0.80	0.80	0.18	0.20	0.24	0.60
F1 * F4	F	5.61	0.10	0.69	5.28	1.90	1.96	0.78
	Significance	0.01	0.91	0.51	0.01	0.15	0.15	0.46
F2 * F3	F	0.84	0.06	3.13	7.05	1.24	2.53	3.27
	Significance	0.36	0.82	0.08	0.01	0.27	0.11	0.07
F2 * F4	F	9.97	1.08	3.59	2.36	3.34	0.55	0.10
	Significance	0.00	0.30	0.06	0.13	0.07	0.46	0.75
F3 * F4	F	0.04	0.01	2.07	1.30	1.15	0.00	4.25
	Significance	0.85	0.93	0.15	0.26	0.29	0.96	0.04
F1 * F2 * F3	F	0.69	0.77	0.29	1.09	0.11	2.88	3.76
	Significance	0.41	0.38	0.59	0.30	0.74	0.09	0.06
F1 * F2 * F4	F	4.66	0.00	0.21	4.43	0.54	0.07	0.49
	Significance	0.03	0.98	0.65	0.04	0.47	0.79	0.49
F1 * F3 * F4	F	0.56	0.01	0.43	4.62	0.42	0.09	4.89
	Significance	0.46	0.95	0.51	0.03	0.52	0.76	0.03
F2 * F3 * F4	F	0.00	0.00	3.38	0.23	0.08	1.83	3.43
	Significance	0.95	0.98	0.07	0.64	0.79	0.18	0.07
F1 * F2 * F3 * F4	F	0.06	1.56	0.94	11.21	0.03	2.32	2.34
	Significance	0.81	0.21	0.34	0.00	0.88	0.13	0.13

* Significance level of 0.05; F1: Land use; F2: Soil type; F3: Road density; F4: Distance from urban center.

## Supplementary Materials

The following are available online at www.mdpi.com/1660-4601/13/5/510/s1, Figure S1: Road distribution in the study area, Table S1: The results of ANOVA for different soil type in the agricultural soil.
