# Supplementary material for: Which Factors Determine Metal Accumulation in Agricultural Soils in the Severely Human-Coupled Ecosystem?
Source: Int J Environ Res Public Health. 2016 May 17;13(5):510. doi: 10.3390/ijerph13050510 (PMC4881135; doi:10.3390/ijerph13050510)
Supplement: Supplementary file 1 [file ijerph-13-00510-s001.pdf]

Supplementary Materials: Which Factors Determine Metal Accumulation in Agricultural Soils in the Severely Human-Coupled Ecosystem?

Li Xu, Shanshan Cao, Jihua Wang and Anxiang Lu

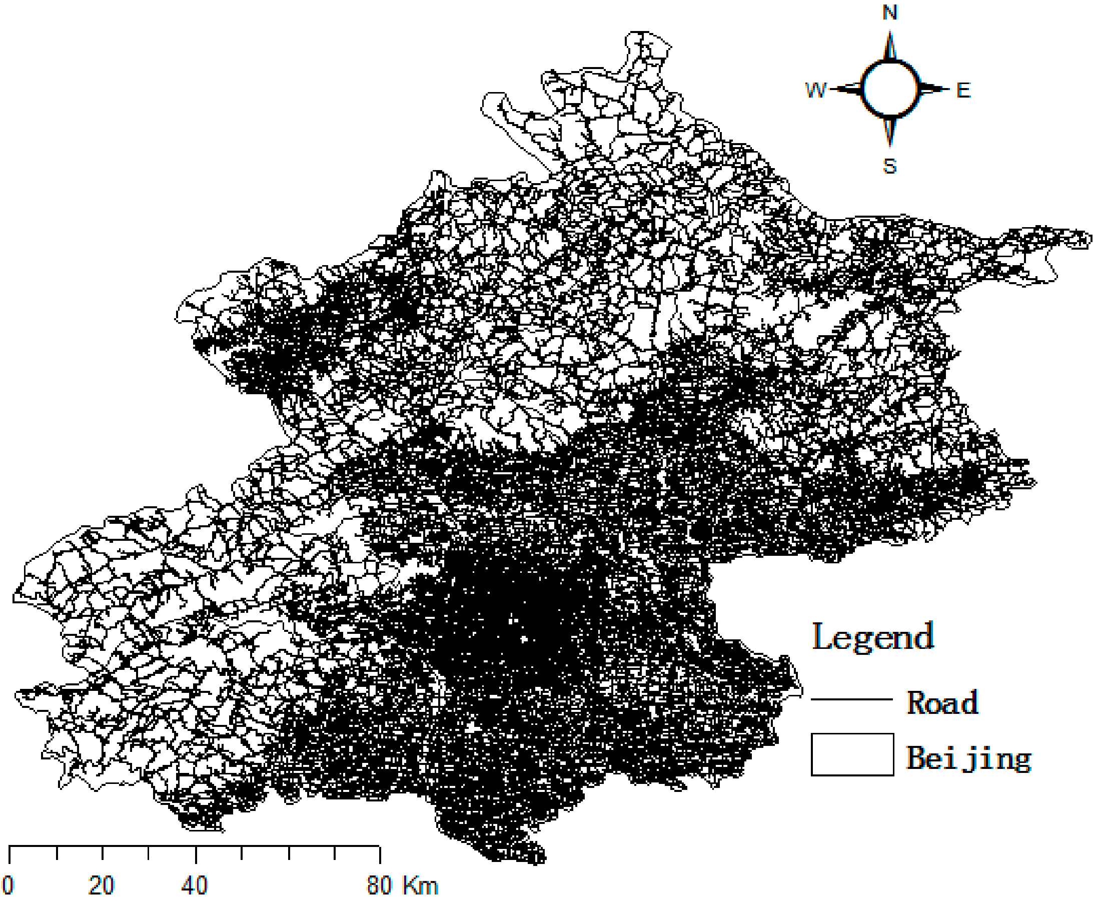

Figure S1. Road distribution in the study area.

Table S1. The results of ANOVA for two soil types (CCS and FAS) in the agricultural soil.

	As	Cd	Cu	Hg	Ni	Pb	Zn
F	0.60	3.58	3.07	2.96	0.48	1.11	0.75
Significance	0.44	0.06	0.08	0.09	0.49	0.30	0.39

CCS: calcareous cinnamon soil; FAS: fluvo-aquic soil.

© 2016 by the authors; licensee MDPI, Basel, Switzerland. This article is an open access article distributed under the terms and conditions of the Creative Commons by Attribution (CC-BY) license (http://creativecommons.org/licenses/by/4.0/).
